# Making “Joy Pie” to Stay Joyful: Self-Care Interventions Alleviate College Students’ Mental Health Challenges

**DOI:** 10.3390/ijerph20053823

**Published:** 2023-02-21

**Authors:** Bu Zhong, Lola Xie

**Affiliations:** 1 Department of Interactive Media, School of Communication, Hong Kong Baptist University, Hong Kong, China; 2 Donald P. Bellisario College of Communications, Pennsylvania State University, University Park, PA 16802, USA

**Keywords:** mental health, college student, self-care, COVID-19, social comparison, emotion as information

## Abstract

As more college students are facing mental health challenges, it is imperative to explore innovative ways of improving their mental health, including developing self-care interventions that help mitigate their stressors. Based on the Response Styles Theory and self-care conceptions, this study creates the “Joy Pie” project that consists of five self-care strategies, aiming to regulate negative emotions and increase self-care efficacy. Using an experimental design and two-wave data collected from a representative sample of Beijing college students (*n*_1_ = 316, *n*_2_ = 127), this study assesses the effects of the five proposed interventions on the students’ self-care efficacy and mental health management. The results show that self-care efficacy helped improve mental health through emotion regulation, which is mediated by age, gender, and family income. The promising results support the effectiveness of the “Joy Pie” interventions in strengthening self-care efficacy and improving mental health. This study offers insights into building back better mental health security among college students at this critical time when the world is recovering from the COVID-19 pandemic.

## 1. Introduction

College students’ anxiety, depression, and other mental health disorders were growing concerns in many countries even before the COVID-19 pandemic hit the world [1,2]. The three-year-long COVID-19 pandemic has been a very stressful period for college students worldwide, as their mental health has been severely exacerbated by the pandemic conditions [3,4]. A great deal of work has been devoted to identifying mental health challenges that college students experienced during the pandemic [5], including college students in China [6], in India [7], and in the United States [8]. Given that college students are less likely to seek professional help under stress, Internet-based interventions provide great resources for students to perform self-care themselves. The existing literature on web- or mobile-based psycho-interventions tends to focus on providing self-care and therapy resources online for students who are experiencing chronic mental health conditions, with the goal to make psychological services more accessible for those who do not have access to offline facilities. Less is known about how to increase students’ self-care efficacy, making them more likely to utilize self-help resources online. Moreover, as past research has been primarily conducted among students with existing mental health conditions, there is limited knowledge about how to motivate college students with no history of mental health problems to practice self-care and utilize available resources for better mental health management.

Moving beyond identifying mental health issues and providing self-care resources online for college students, this study proposes a mobile communication-based intervention with five strategies for improving college students’ self-care efficacy and mental health that is based on the Response Styles Theory [9]. Drawing on previous studies on coping strategies, the intervention was designed as the five-step “Joy Pie” project that consists of five strategies: (1) delay worries; (2) talk with someone; (3) be less critical; (4) design a to-go strategy for yourself, and (5) weekend reflection. As students practice all five strategies in a row, they make a “Joy Pie” as a way to mitigate school-related stressors, helping them stay joyful. The goal is to empower college students by building up their self-care reliance for coping with campus stressors. Using an experimental design, this research assesses the effectiveness of the “Joy Pie” self-care strategies; the findings shine a light on mental health self-care among college students, particularly by regulating negative emotions such as stress, anxiety, and depression.

## 2. Literature Review

Among China’s 28 million college students [10], one in every four (24.6%) has experienced depression and other mental health problems [11]. The COVID-19 pandemic further added to the complexity of students’ mental health issues, which has become a social and public health concern in China [6]. A large-scale longitudinal study associated the pandemic with a rise in depressive and anxiety symptoms in Chinese college students, including acute stress, anxiety, and depression [12]. It also found that about 10% of nearly 70,000 Chinese college students under study showed persistent or developed new mental health problems during the COVID-19 pandemic. Youth mental health issues are well documented to be directly associated with impairments in academic success and personal relationships, leading to social withdrawal, self-harm, and even suicidality [13]. The importance of prevention and intervention strategies has been recognized as a means of addressing youth mental health needs, especially in reducing stress, anxiety, and depression [14]. The enormous size of China’s youth population with mental health problems highlights the need for creating self-care interventions to improve mental health management.

### 2.1. The Response Styles Theory

Assessing the plausibility of non-medical interventions for improving college students’ mental health requires us to apply novel theoretical approaches to understand their vulnerability to mental health challenges. This study employs the Response Styles Theory of Depression [9,15] to study how youths try to regulate emotions, treating it as a theoretical framework to guide our experimental design for measuring the effectiveness of our proposed intervention strategies. The Response Styles Theory proposes two major responses to depression: distraction and rumination [15]. Rumination refers to the process of individuals focusing on their own experiences, thoughts, and feelings, especially “thinking perseveratively about one’s feelings and problems” ([16], p. 400). Nolen-Hoeksema argues that there are three major mechanisms involving rumination. First, rumination intensifies the depressed mood upon thinking, making it more likely that people will use the negative thoughts and memories activated by their depressed mood to understand their current circumstances. Second, rumination interferes with effective problem solving, in part by making one’s thinking more pessimistic. Third, rumination interferes with instrumental behavior, leading to increases in stressful circumstances [16].

After analyzing over 220 studies that examined maladaptive forms of self-focused attention in people prone to depression, anxiety, or other forms of mental issues, Mor and Winquist [17] found that rumination was the form most strongly and consistently related to depressive symptoms and depression disorders. After controlling for depression levels, research has repeatedly found that rumination is closely associated with certain negative cognitive styles such as hopelessness, pessimism, perfectionism, self-criticism, and neuroticism [16,18]. Rumination, as a special kind of self-focus, could mediate, partially or fully, the relationship between major depressive episodes and risk factors such as maladaptive attitudes and negative cognitive styles [19]. Those who use rumination as a response to dysphoria could experience more intense episodes of depression [9].

The Response Styles Theory states that an alternative for regulating depressive emotions is to use pleasant or neutral distractions to regulate negative feelings such as stress, anxiety, or a depressed mood. Such distracting responses, as a part of emotion regulation, are found to be able to divert people’s attention away from depressive ideas and consequences, allowing them to refocus on pleasant or positive ideas and activities [16]. In many cases, persistent and intense episodes of depression could easily lead to worse consequences, including suicidal thoughts. Suicidal thoughts, as a negative emotion, inhibit the ability to readjust health behavior to cope with stressors or mental health challenges. Moreover, Nolen-Hoeksema and her colleagues [16] emphasize that effective distractions do not include inherently dangerous or self-destructive activities, such as heavy drinking, drug abuse, or aggressive behavior. Such high-risk distractions may temporarily take attention away from current stressors in the short-term, but could be harmful in the long run [16]. Thus, this theory and the conceptions of self-care efficacy are an appropriate theoretical framework for guiding the present study.

### 2.2. Self-Care Efficacy and Self-Care Intervention

A promising alternative to improve mental health is to utilize interventions that allow youths to administer self-care. Self-care interventions are highly cost-effective and can help maximize youths’ autonomy by decreasing their reliance on mental health professionals [20,21]. Existing research associates self-care interventions with reduced depression, anxiety, and stress among college students [22]. However, relatively few self-care intervention programs have been implemented in China, and their effectiveness remains poorly understood among Chinese students. Considering the effectiveness of online interventions used for U.S. college students [23], it is imperative to see how online interventions may help Chinese students cope with mental issues. An individual with high self-care efficacy is expected to exhibit increased adherence to activities that promote health and decreased psychological symptoms. Other studies support a positive relationship between self-care efficacy and quality of life, and a negative correlation with negative moods [24].

Self-efficacy has been identified as a prerequisite for behavioral change in patients with chronic illness [24]. An individual’s sense of self-efficacy can provide the foundation for motivation, well-being, and personal accomplishment. Self-care self-efficacy, which will be referred to as “self-care efficacy” hereafter for brevity, is defined as an individual’s confidence in being able to perform relevant self-care behaviors in a given situation [25]. An individual with high self-care efficacy is found to exhibit increased adherence to self-care activities [26]. Studies support a positive relationship between self-care efficacy and life satisfaction [27], and between self-care efficacy and positive health outcomes, whereas self-care efficacy has a negative correlation with negative moods [24].

For instance, among cancer patients, a high degree of self-care efficacy was found to be significantly associated with decreased physical and psychological symptoms [28]. In diabetes mellitus patients, self-care efficacy has been found to be the most important predictor of self-care behaviors, as well as acting as a clinical pathway through which diabetes care could be improved [29].

Therefore, this study first explores how self-care efficacy may influence Chinese college students’ experience of negative emotions and mental health related to major campus stressors involving personal relationships and schoolwork pressures. Drawing on previous studies, we propose:

**Hypothesis 1.**  *College students’ self-care efficacy is negatively associated with the presence of their negative emotions*.

**Hypothesis 2.**  *College students’ self-care efficacy is positively associated with their mental health conditions*.

In recent years, self-care interventions have been found to be a good first step in managing college students’ mental health issues [30]. While formal psychological treatments for college students’ mental disorders are effective, many students tended to delay or avoid seeking professional help because of system-related barriers (e.g., long waiting list) and attitudinal barriers (e.g., embarrassment or stigma) [31]. Given these barriers, delivering evidence-based interventions to provide help with stress management in a self-care format might be a more practical and psychologically acceptable approach [32]. Because of the features of scalability at a low cost and time flexibility, self-care interventions have the potential to overcome system-related and attitudinal barriers [32].

**Figure 1:**
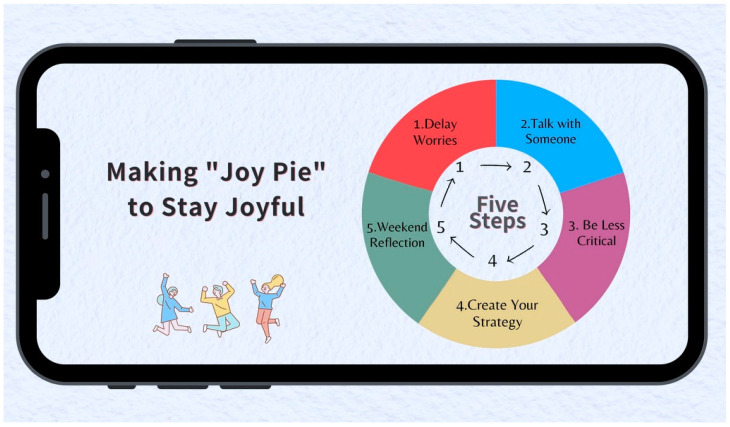
The “Joy Pie” interventions for mental health management (Illustration by Lola Xie).

This study develops a self-care intervention, the “Joy Pie” intervention (Figure 1), to help college students improve their mental health condition through self-care. Different from regular interventions in which participants receive the treatment with the help of a professional guide or therapist [33], self-care interventions involve personal coping strategies and are informed by conceptions of self-care that emphasize personal autonomy, resilience, self-efficacy, self-control, self-actualization, and self-stewardship [34]. As a supplement to other interventions, self-care interventions can be easily self-administered by individual users at their own pace and in an environment where they feel relaxed, which is critical to improve one’s mental health [35].

### 2.3. Coping Strategies and Mental Health

Extensive research has been carried out on how individuals cope with stress, highlighting the correlation between the management of stressful life events and mental or physical health [36]. Results from previous studies indicate that the existence of stress originating from stressful life events may be less important to mental health than how an individual copes with stress [37]. Different strategies have been identified depending on the conceptualization of coping, and yet, only some of them have been repeatedly examined and tested before. Based on prior studies, we proposed the “Joy Pie” project consisting of five self-care interventions.

Among the optional strategies, distraction and avoidance behaviors have been found to be the most common forms of coping mechanisms [38], although there are still unresolved issues concerning the effects. While some studies related the escape-avoidance strategy with an increase in psychological distress and depression [39], some others associated it with positive outcomes, especially in uncontrollable situations [36,40]. In a study on the coping mechanisms of healthcare workers during the COVID-19 pandemic in Japan, the authors identified that over 70% of study participants adopted the escape-avoidance strategy for coping with stress [36]. While this strategy was positively associated with depression at low levels of stress, it has been associated with more adaptive functioning of these outcomes at higher levels of stress [40], which means that in a given situation with a high level of restrictions and strong external pressure, an evasive strategy may have been the best way to maintain mental health. Drawing on the literature, we proposed an evasive strategy of “delay worries”, as we expected our participants to be facing high levels of pressure as they had to manage relationship and schoolwork stresses while their schools went into lockdown from time to time under the zero-COVID policy in China.

Another coping strategy that has been frequently adopted is communication, which also involves relying on people for social support in times of stress [41,42]. The ability to communicate both verbally and non-verbally about how one feels has been associated with positive health outcomes [42]. In a study on high-achieving students in high school [41], family communication was negatively correlated with perceived stress and positively associated with global life satisfaction, suggesting that family communication may be a more adaptive coping strategy for high-achieving students. Family communication also emerged as the strongest predictor of school functioning. By talking to others about the stresses and negative emotions, one can also mobilize both information and emotional support that can ease anxiety and relieve emotional distress [36]. Therefore, we encouraged our participants to talk to others about their stresses and anxiety by proposing the strategy of “initiate a chat”.

Meta-analytic findings have consistently related self-criticism and blame to depressive symptoms and poor health [43,44]. Self-criticism is a reflexive psychological behavior elicited when individuals are unsatisfied with the acquired outcome of important decisions or experience crucial failures [45,46]. Multiple studies have associated increased levels of self-criticism with increased levels of depression [45]. In response, self-forgiveness has been proposed as an emotion-focused coping approach to dealing with stresses that result from personal failure, guilt/shame, or general incongruence between personal values and actual behavior [47]. Self-forgiveness involves reducing negative, and increasing positive, thoughts and emotions regarding oneself [48]. The use of self-forgiveness as a coping strategy is expected to encourage adaptive coping including self-care, lessen stressors, and alleviate mental health problems [43]. Thus, we proposed the strategy of “be less critical” to encourage our participants to engage in self-forgiveness.

Meanwhile, meditation practices can form a key component of coping strategies for mitigating anxiety and stress [49]. As a non-pharmacological self-management solution, meditation practices aim at training one’s self-awareness of the present experience, including one’s thoughts, feelings, and sensations without any judgment, filter, or expectations [50]. It has been used widely for the reduction of stress and the promotion of health, and the results broadly support the beneficial effects of such practices on physical and mental health, as well as on cognitive performance [51]. Based on the existing literature, we proposed “weekend reflection” to enhance the participants’ interoceptive awareness of their emotional and mental phenomena [52]. In addition, previous studies also suggest that the same strategy can be effective or ineffective in different situations, as each problem or situation requires the use of a specific coping strategy [53]. Additionally, it is almost impossible to include the entire universe of potential coping strategies in only one study. The effectiveness of self-care strategies could vary between people in different situations, and even vary for the same person at different times. Therefore, we also encouraged our participants to create their own self-care strategies to capture the potentially wide variety of strategies that could be incorporated by the participants and be sensitive to situational differences.

### 2.4. The “Joy Pie” Interventions

The “Joy Pie” interventions include five strategies: (1) delay worries; (2) initiate a chat with others; (3) be less critical; (4) create one’s own self-care strategies; and (5) weekend reflection (see Figure 1). They were developed by drawing on the literature of self-care and coping strategies [25,34,54,55]. Informed by conceptions of self-care that emphasize personal autonomy, resilience, self-efficacy, self-control, self-actualization, and self-stewardship [34], the “Joy Pie” project encourages self-care reliance in managing one’s mental health conditions. Here, self-care is defined as the actions that individuals take to enhance, restore, or maintain health; prevent or limit illness; and cope with illness with or without the support of healthcare professionals [54,56]. A systematic review found that a very small number of studies concerning the improvement of individual self-care capabilities actually measured self-care capability changes [57], which highlights the importance of our study. Based on the literature reviewed, the following research questions were formulated to evaluate the effects of the proposed intervention strategies:

**RQ1.** How do the “Joy Pie” interventions affect college students’ self-care efficacy?
**RQ2.** How do the “Joy Pie” interventions affect college students’ experience of negative emotions and mental health when facing relationship and schoolwork pressures?

## 3. Method

### 3.1. Participants and Procedures

College students in Beijing universities were recruited in two waves in June and July 2021 on Jishuyun (https://www.databnu.com/#/index, accessed on 20 December 2022), a Beijing-based online panel platform. In the panel, there were over 100,000 students from 92 universities and colleges in Beijing, which can be taken as representative of all college students in Beijing. For this study, Jishuyun randomly sent survey invitations to participants within the panel, who were registered and confirmed as students from Beijing colleges. Participants who completed the initial survey were then randomly assigned to either an experimental condition—requiring them to practice the five “Joy Pie” interventions for four weeks—or a control group—reporting only their mental health conditions twice without practicing the interventions. A total of 316 students completed the initial survey (T1) in June 2021 when they were at school, and 127 of them completed the second-wave survey (T2) in July 2021. The second wave recruited fewer students due to the start of their summer vacation. Electronic informed consent was then obtained online from the participants, who were informed that they could withdraw from the study at any time.

### 3.2. The Procedure

This study consisted of two parts: (1) through an online survey, it explored how major predictors, including self-care efficacy and negative emotions such as depression, anxiety, and stress, may affect Chinese college students’ mental health conditions; and (2) using an experimental design, this study assessed the effectiveness of the proposed intervention strategies in improving mental health conditions. As shown in Figure 1, the intervention program consists of five self-care strategies. Detailed practice instructions were provided about each of the strategies. During the weekdays, the students were asked to:

*“(1) Delay worries—setting a time for those worries later, if you have to; (2) Initiate communication with others; (3) Try to be less critical of yourself and others; (4) Develop some self-care tactics and try to use them daily, e.g., jogging, listening to music or podcast, playing with a pet, chatting with a friend, etc.”*

On the weekends, they were asked to designate 30–60 min for self-reflecting on stressors experienced and how to overcome them. If no major stressors occurred in a particular week, they were asked to meditate by taking deep breaths for 30–60 min. The self-care interventions were presented as a color image so that they could be easily disseminated and saved to a smartphone.

### 3.3. Survey Design and the Pre-Test

The participants completed a survey at T1 and responded to validated measures including self-care efficacy, the 21-item Depression Anxiety Stress Scale (DASS-21), and mental health. The survey was designed in English, and the stimulus scenarios and measures were later translated into Chinese by two of the authors whose native language is Chinese. The authors discussed the appropriateness of the translation and pilot tested the survey with a small sample (*N* = 24) prior to data collection for the primary study. In the pre-test, the top stress sources for Beijing college students were identified as being school-related, e.g., school workload and academic performance, and relationship-related, e.g., their relationships with schoolmates, professors, and family members.

After removing the data that failed the quality check (i.e., minimum time spent on the survey and straightlining responses), the final dataset included responses from 316 college students at T1. The average age of the students was 20.65 years old (*SD* = 1.77). A majority of them were females (61.7%). Participants reported their self-perceived family income: 69.3% were middle income, 29.7% low income, and 0.9% high income. Among the participants, 39.6% were from big cities, 47.8% from medium-sized cities, and 12.7% from small towns or rural areas. Participants also reported their parents’ education levels: 38% of their parents received an education lower than high school, 25.0% a high school diploma, 35.1% a college degree, and 1.9% a master’s or doctorate degree.

### 3.4. Experiment Design

The students who participated in the T1 survey were randomly assigned into either an experimental or a control group. For the control group, participants were invited to take a follow-up survey four weeks later (T2). The survey questions at T2 were the same as those at T1, which measured any changes in their self-care efficacy, experience of negative emotions, and mental health conditions. For the experimental group, participants were reminded over the following four week to practice the “Joy Pie” self-care interventions through a weekly message, which asked them to try to manage any stressors they experienced in life. Four weeks later, they were invited to take a follow-up survey and report their self-care efficacy, experience of negative emotions, and mental health conditions at T2. They were also asked to report their practice frequency and evaluate their perceived usefulness of the “Joy Pie” intervention strategies. After removing data that failed the quality check (i.e., minimum time spent on the survey and straightlining responses), the final dataset containing 127 responses from Beijing college students at T1 and T2 were analyzed.

### 3.5. Measures

#### 3.5.1. Self-Care Efficacy

Self-care efficacy was operationalized as the extent to which respondents felt confident in their ability to carry out specific self-care behaviors in the face of their stressors. The 7-item measure adapted from previous research [25] was used to measure respondents’ self-care efficacy. Students were asked to rate their agreement level to seven statements on a 5-point Likert scale from 1 (strongly disagree) to 5 (strongly agree) (see Appendix A). The items have a good reliability with higher scores, indicating higher efficacy (*α* = 0.74).

#### 3.5.2. Negative Emotions

The widely adopted Depression Anxiety Stress Scale-21 (DASS-21) [58] was used to measure respondents’ experience of negative emotions. Considering the cultural sensitivity of the topic, we took out one item from the original scale (i.e., “I felt I wasn’t worth much as a person”). Seven items were used to measure respondents’ stress level (e.g., “I found it hard to wind down”), seven items were used to measure their anxiety level (e.g., “I was aware of dryness of my mouth”), and six items were used to measure their depression level (e.g., “I couldn’t seem to experience any positive feeling at all”). Students were asked to rate to what extent the statements applied to them from 1 (did not apply to me at all) to 4 (applied to me very much) (see Appendix A). Together, the 20 items formed a reliable index, *α* = 0.89, with higher scores indicating higher levels of symptoms.

#### 3.5.3. Mental Health Indicator

Findings from the pilot study indicated that most respondents’ stressors mainly originated from two aspects: personal relationships with others, such as friends, and schoolwork pressures. Therefore, two scenarios were presented to the participants to measure their mental health when facing relationship and schoolwork stressors. Eight items from a previous study [59] were used to measure their mental health when facing relationship stressors (Scenario 1) and schoolwork pressures (Scenario 2). Respondents were asked to indicate how they would agree to the 8 statements in the face of the stressors as described in the scenario on a 5-point Likert scale from 1 (strongly disagree) to 5 (strongly disagree) (See Appendix A). Both measures of relationship-related mental health (*α* = 0.83) and schoolwork-related mental health (*α* = 0.88) were reliable, with higher scores indicating better mental health under stress.

### 3.6. Practice Frequency and Intervention Effectiveness

Respondents in the experimental group were asked to report their frequency of practicing the intervention strategies since the T1 survey, which served as a manipulation check of the experiment as students were instructed to practice the strategies on their own. Practice frequency was measured with the item: “In the past weeks, how often did you practice the Joy Pie strategies?” The item was measured on a 5-point Likert-type scale (1 = none or not much to 5 = very often/everyday). In addition, respondents in the experimental group were also asked to report the perceived helpfulness of each of the intervention strategies. They were also asked to report their perceived improvements in different aspects of their life after practicing the “Joy Pie” interventions, including overall improvements, health condition improvements, and improvements in schoolwork and personal relationships. The items were measured on a 5-point Likert scale, with higher scores indicating higher levels of improvement. The items used are presented in Appendix A.

### 3.7. Statistical Analysis

We first ran descriptive statistics on all of our variables for both waves of the surveys, as well as on the data on students’ self-report practice frequency and perceived helpfulness of the intervention program. To test our first two hypotheses, we ran a correlation test among all studied variables, including self-care efficacy, negative emotions, and mental health scores. Then, we performed hierarchy regressions on both negative emotions and mental health scores to examine whether self-care efficacy plays a role in alleviating negative emotions and improving mental health. Next, we further examined the effectiveness of the “Joy Pie” intervention using a hierarchy regression to analyze self-care efficacy against practice frequency, answering RQ1. Lastly, we used paired samples *t*-tests to evaluate how students’ self-reporting of negative emotions and mental health changed after the intervention.

## 4. Results

Descriptive statistics for self-care efficacy, negative emotions, relationship-related mental health, and schoolwork-related mental health in both waves of the survey are presented in Table 1.

Meanwhile, we also ran a descriptive analysis of students’ practice frequency and perceived helpfulness of the “Joy Pie” interventions. The analysis shows that for every strategy, the average helpfulness score was between 3.68 and 3.06 out of 5, indicating that the respondents considered the interventions to have moderate to high levels of effectiveness. Among the five strategies, Strategy 2, “initiate a chat with others”, was considered the most effective (*M* = 3.76, *SD* = 0.92), followed by Strategy 4, i.e., “create one’s own self-care strategies” (*M* = 3.68, *SD* = 1.05), and Strategy 5, “weekend reflection” (*M* = 3.69, *SD* = 1.13). The average score for Strategy 3, “be less critical”, was 3.53 (*SD* = 0.94). Strategy 1, “delay worries”, was considered the least helpful (*M* = 3.06, *SD* = 1.14).

They were also asked to report their perceived improvements in different aspects of their life after practicing the “Joy Pie” interventions, including overall improvements (*M* = 3.80, *SD* = 0.67), health condition improvements (*M* = 3.83, *SD* = 0.71), and improvements in schoolwork (*M =* 3.60, *SD* = 0.93) and personal relationships (*M =* 3.34, *SD* = 1.06). The average scores were above 3.5 out of 5 except for personal relationships, indicating moderate levels of effectiveness of the intervention in the aspects mentioned above. Then, we tested each hypothesis and answered the research questions using inferential statistics, as shown in the next subsection.

### 4.1. Associations between Self-Care Efficacy, Negative Emotions (H1), and Mental Health (H2)

H1 proposed that self-care efficacy would be negatively associated with college students’ experience of negative emotions. H2 proposed that self-care efficacy would be positively associated with their mental health while facing stressors induced by relationships and schoolwork pressures. The associations were first examined by calculating Pearson’s correlation coefficient. The results indicated that self-care efficacy was negatively associated with negative emotions, and positively associated with students’ mental health when facing both relationship-related and schoolwork-related stresses. See Table 2 for the study variables’ Pearson’s correlation coefficients.

The hierarchical regression analyses were then conducted. Control variables including age, gender, family household income, geolocation, and parents’ education were entered as the first step of each analysis to control their potential effects on mental health wellness. In the second step, self-care efficacy was entered to examine its main effects.

For H1, variables in step one of the regression model accounted for a non-significant 3% of the variance in students’ negative emotions (*F*(5, 315) = 1.72, *p* = 0.13). Results show that none of the demographic variables affected respondents’ experience of negative emotions. In step two, the main effects of self-care efficacy explained an additional 29.7% increment in the variance in the experience of negative emotions (*F*(6, 315) = 24.68, *p* < 0.001). The regression weights from the full six-variable model demonstrated that self-care efficacy was negatively associated with negative emotions (*B* = −0.56, *t* = −11.65, *p* < 0.001). This suggests that participants with higher self-care efficacy reported fewer experiences of negative emotions. Therefore, H1 is supported. The detailed results are reported in Table 3.

For H2, the variables in step one of the regression model measuring relationship-related mental health accounted for a significant 5.5% of the variance in mental health (*F*(5, 315) = 3.62, *p* < 0.01). Results show that none of the demographic variables, except age, affected respondents’ relationship-related mental health (*B* = 0.16, *t* = 2.80, *p* = 0.005). The older students had significantly better mental health in face of relationship-related stressors than their younger counterparts. None of the other demographic variables significantly influenced students’ mental health when facing relationship-related stressors.

In step two, the main effects of self-care efficacy explained an additional 25.4% increment in the variance in relationship-related mental health (*F*(6, 315) = 23.05, *p* < 0.001). The regression weights from the full six-variable model demonstrated that self-care efficacy was positively associated with relationship-related mental health (*B* = 0.52, *t* = 10.66, *p* < 0.001). This suggests that participants with higher self-care efficacy reported better mental health in the face of relationship stressors. Meanwhile, students’ age remained a significant positive predictor of their mental health when dealing with relationship stresses (*B* = 0.10, *t* = 2.02, *p* < 0.05). Students’ parents’ educational level also emerged as a significant positive predictor of their mental health when dealing with relationship-related stressors (*B* = 0.10, *t* = 2.07, *p* < 0.05). This means that students with parents who had higher educational levels exhibited better mental health when facing relationship stressors (see the detailed results in Table 4).

As shown in Table 5, in step 1 of the regression of schoolwork-related mental health, the control variables accounted for a significant 3.9% of the variance in mental health (*F*(5, 315) = 2.50, *p* = 0.03). Results show that among the demographic variables, age affected respondents’ schoolwork-related mental health (*B* = 0.11, *t* = 2.04, *p* = 0.04). This means that older respondents had better mental health in the face of schoolwork-related stressors compared to their younger counterparts. In Step 2, the main effects of self-care efficacy explained an additional 20.6% increment in the variance in schoolwork-related mental health (*F*(6, 315) = 16.72, *p* < 0.001). The regression weights from the full six-variable model demonstrated that self-care efficacy was positively associated with schoolwork-related mental health (*B* = 0.46, *t* = 9.19, *p* < 0.001). This means that participants with higher self-care efficacy reported better mental health in the face of schoolwork stressors. Thus, H2 is supported. The detailed results are reported in Table 5.

### 4.2. Effects of the “Joy Pie” Intervention on Self-Care Efficacy (RQ1)

RQ1 asked how the “Joy Pie” interventions affect college students’ self-care efficacy. A hierarchical regression analysis was conducted to address RQ1. The result indicates that, after controlling for the effects of demographic variables and self-care efficacy at T1, the main effect of practice frequency explained an additional 3.6% increment in the variance in schoolwork-related mental health (*F*(7, 119) = 7.77, *p* < 0.001). The regression weights from the full seven-variable model demonstrated that practice frequency was positively associated with self-care efficacy (*B* = 0.19, *t* = 2.50, *p* = 0.01). This means that practicing the “Joy Pie” interventions helps enhance college students’ self-care efficacy. The detailed results are reported in Table 6.

### 4.3. Effects of “Joy Pie” Interventions on Negative Emotions and Mental Health (RQ2)

RQ2 asked how the “Joy Pie” interventions affect college students’ experience of negative emotions and mental health. To answer the research question, paired samples *t*-tests were performed to compare the means at T1 and T2 for both the control and experimental groups. Results show that for the experiment group, there was a significant decrease in negative emotions from T1 (*M* = 2.88, *SD* = 0.59) to T2 (*M* = 2.61, *SD* = 0.73, *p* = 0.009) after four weeks of practicing the intervention strategies. There was a significant improvement in both relationship-related and schoolwork-related mental health. Students’ relationship-related mental health improved significantly from T1 (*M* = 3.53, *SD* = 0.80) to T2 (*M* = 3.78, *SD* = 0.68, *p* = 0.04). There was also a significant improvement in schoolwork-related mental health among the respondents from T1 (*M* = 3.46, *SD* = 0.77) to T2 (3.76, *SD* = 0.70, *p* = 0.008). The detailed results are reported in Table 7.

In comparison, the results from the control group show no significant difference in any of the study variables at T2 compared to T1 (Table 8). Overall, the results suggest the effectiveness of our intervention strategies in strengthening the respondents’ self-care efficacy, and as a result, in improving their mental health. Table 7 and Table 8 report the paired differences of the study variables for each group.

## 5. Discussion

This study, guided by the Response Styles Theory, conceptions of self-care, and coping strategies published in the literature, proposes the “Joy Pie” intervention consisting of five strategies for college students to manage life stresses and improve their mental health management. Results indicated the effectiveness of the self-care strategies among a group of college students in Beijing after practicing them for four weeks. The findings concerning the benefits of self-care strategies add fresh evidence to the importance of embedding the methods and concepts from humanities and social sciences into caring for youth mental health [60].

Consistent with the findings from the West [17,61], this study shows that self-care efficacy is negatively related to Chinese students’ experience of negative emotions, including depression, anxiety, and stress, and is positively related to mental well-being in the face of relationship-related and schoolwork-related stressors. It also shows the role of age, gender, and parent education in managing such campus stressors. Older college students maintained better mental health than their younger peers when facing relationship-related stressors. Young men had significantly better mental health in the face of schoolwork-related stressors than their female counterparts, which is consistent with the findings reported in previous studies [62]. The students with parents who had received a higher education reported better mental health that their peers when facing the same relationship-related stressors, suggesting that the latter might have limited resources to deal with stressors [63]. However, no significant association was detected between mental health and the students’ hometown location, regardless if they were from big cities, medium cities, or small towns/rural areas.

The data show that college students generally reported the “Joy Pie” interventions to have a moderate to high effect on their mental health management. Among the five strategies, Strategy 4, or “Create your own self-care strategies,” was considered most effective, followed by Strategy 5—“weekend reflection,” Strategy 2—“Initiate a chat with others”, and Strategy 3—“Be less critical”, with the last being Strategy 1—”Delay worries”. Specifically, the college students reported that the more they practiced the “Joy Pie” strategies, the better they felt in their daily activities and the better personal relationships they had with others around them. The use of the “Joy Pie” interventions also contributed to stronger self-care efficacy after controlling for the level of such efficacy at T1, showing the effectiveness of our strategies in improving participants’ confidence in being able to perform relevant self-care behaviors in a given situation [25]. Since self-care efficacy has been identified as a prerequisite for behavioral change in patients with chronic illness [24], our strategies are expected to enhance participants’ motivation to adhere to self-care activities [26].

Comparing the data collected at T1 and T2, the analyses discovered that the college students’ mental health related to campus stressors such as relationships and schoolwork improved significantly after the four-week practice of the “Joy Pie” project. However, it might be possible that if they could practice the strategies for longer than four weeks, some differences might emerge in future studies. Overall, the results suggested the effectiveness of our intervention strategies in strengthening college students’ self-care efficacy, leading to improved mental health.

### 5.1. Theoretical Implications

Theoretically, the current research has expanded the previously proven significance of self-care efficacy by adding new evidence that relates to youth mental health management. The finding of a negative relationship between self-care self-efficacy and negative emotions supports the theoretical proposition that self-efficacy is a cognitive factor associated with the development of depressive symptoms [25]. It also adds to the empirical evidence in support of the positive relationship between self-care efficacy and mental health [24]. This finding is important because there is a strong consensus that knowing what one should do for his/her health does not necessarily mean that self-care behaviors will follow [64]. An individual makes judgments about his/her capacity to engage in self-care behaviors to produce desired outcomes, and it is reasonable to hypothesize that students would be more likely to adhere to self-care behaviors if they are more confident in their ability to carry out these behaviors. The conception of self-care efficacy provides a bridge between knowledge and actual self-care behaviors [64].

Our findings also provide empirical support for the effectiveness of self-care approaches in preventing or alleviating the high stress and associated mental problems among college students. Previous studies found that mental health effects can be different depending on the selected stress coping strategy [36]. Among the five “joy pie” strategies, strategy 2, “initiate a chat with others”, was considered the most effective. Students coping by communicating with and relying on important people around them are found to have fewer mental health problems [41]. Communication appears to be an effective way to contend with life stresses in health situations. Strategy 4, “create one’s own self-care strategies”, was considered the second most effective coping method. Encouraging participants to create their own self-care strategies may induce a feeling of control [65]. People who are confident in their abilities and engage in activities that promote health are also expected to adhere to such activities. Engaging in activities that they personally feel comfortable with may give them greater confidence and enhance their self-care efficacy, thus reducing their risk of developing negative emotions.

In addition, strategy 5, “weekend reflection”, and strategy 3, “be less critical”, were considered moderately effective, whereas strategy 1, “delay worries”, was considered the least effective, despite it still being above 3 points out of 5. Scholars hold different opinions about the effectiveness of the escape-avoidance strategy to improve mental health [36,39,40]. Our finding supports there being a moderate effect and highlights the significance of this strategy in a situation with high levels of restrictions and strong external pressures, such as that during the period of the COVID-19 pandemic [66,67]. As the students faced restrictions in their daily experiences to prevent the spread of COVID-19, an evasive strategy may have been be the only way for them to manage negative emotions and maintain mental health. Our finding also highlights the significance of context when analyzing the effectiveness of coping strategies. The use of a coping strategy should be considered in the specific situation, as the same strategy can be effective or not depending on the individual’s perception of the situation as threatening or not [53]. Future studies should also consider the levels of restrictions and external pressures in specific situations while examining the effectiveness of similar strategies.

### 5.2. Practical Implications

Going beyond the prior research in identifying mental health challenges among college students [12,23], this study focused on developing self-care interventions that assist youths in their self-management of mental health. The key findings are that college students could be sensitive to self-care interventions, which could be useful adjunctive treatment options. The finding of a positive relationship between self-care self-efficacy and mental health supports and extends the work of previous investigators who established that self-care efficacy is an important factor for quality of life [25,68]. Clinically, these findings suggest the need for paying close attention to the self-care self-efficacy in college students experiencing stressors and negative emotions.

For college students facing campus stressors, in addition to seeking medications, psychiatric consultations, and therapies from mental health professionals, it is also beneficial to adopt non-medical interventions to manage mental health issues. Lessons and training could also be provided to enhance their self-care efficacy in managing mental health issues during their college years.

### 5.3. Limitations

The findings of the study need to be considered in light of several limitations. First, the study relied on self-report data rather than observational data. Future researchers should consider collecting objective data. Another limitation is that the T2 sample was smaller than the T1 sample due to the difficulty of reaching out to the students when the pandemic became worse in China during those months, which was unexpected for T2, although the dropout rates were comparable to those in previous studies such as 1–50% [69], 2–50% [70], or 0–82% [71]. These considerations should be factored into the interpretation of the results.

On the other hand, self-help may increase willingness to seek professional help among college students [33]. Therefore, innovative methods are worth investigating, such as different ways of increasing adherence and transferring common factors associated with improvements in face-to-face/guided programs into self-guided interventions. As a result, the most accurate strategies can be developed to reach college students who are in need of mental health help, but do not access the relevant resources due to barriers.

## 6. Conclusions

Young adults generally believe that they are entitled to happiness, which is assumed to drop on them automatically without much effort. This study indicates that for college students, it is easier to be stressed or depressed than happy, as they find that the latter requires hard work. For college students, making “Joy Pie,” or practicing self-care strategies, can be a path to happiness. The findings show that increased self-efficacy is associated with decreased negative emotions and better mental health conditions. The concept of self-care efficacy highlights that college students have the capacity to learn about their mental health needs and manage their own mental health. An intervention program that provides self-care efficacy training is warranted, which could lead to increased adherence to treatment, behaviors perceived as promoting health, and decreased physical and psychological symptoms. In addition, the “Joy Pie” strategies were found to be effective at enhancing participants’ self-care efficacy, lessening negative emotions, and improving mental health. Hence, this study provided college students, parents, and health professionals with information concerning self-care efficacy, negative emotion regulation, and mental health management. Overall, this research offers timely health insights into building back better mental health security among college students at this critical time when the world is recovering from the COVID-19 pandemic.

## Figures and Tables

**Table 1:** Descriptive statistics of key variables.

Variables	*M*	*SD*
T1—Self-care efficacy	3.71	0.58
T1—Negative emotions	2.88	0.59
T1—Relationship-related mental health	3.53	0.8
T1—Schoolwork-related mental health	3.46	0.77
T2—Self-care efficacy	3.87	0.60
T2—Negative emotions	2.61	0.73
T2—Relationship-related mental health	2.78	0.68
T2—Schoolwork-related mental health	3.76	0.77

**Table 2:** Bivariate correlation of study variables.

Variable	1	2	3	4
1. Self-care efficacy	–			
2. Negative emotions	−0.57 **	–		
3. Relationship-related mental health	0.54 **	−0.38 **	–	
4. Schoolwork-related mental health	0.48 **	−0.36 **	0.71 **	–

Note: ** *p <* 0.01.

**Table 3:** Hierarchical regression estimates predicting negative emotions.

Variable	Δ*R*^2^	*t*	*B*	*p*
*Step 1*	0.03 ***			
Age		−1.79	−0.10	0.08
Gender		1.47	0.08	0.14
Family income		−1.11	−0.07	0.27
Geolocation		0.76	0.04	0.45
Parent education		−0.55	−0.03	0.58
*Step 2*	0.30 ***			
Age		−0.78	−0.04	0.43
Gender		0.60	0.03	0.55
Family income		−0.26	−0.01	0.80
Geolocation		0.05	0.01	0.96
Parent education		−0.52	−0.03	0.60
Self-care efficacy ***		−11.65	−0.56	0.00

Note: *** *p* < 0.001.

**Table 4:** Factors contributing to mental health while facing relationship pressures.

Variable	Δ*R*^2^	*t*	*B*	*p*
*Step 1*	0.055			
Age *		2.80	0.16	0.005
Gender		−0.86	−0.05	0.39
Family income		0.93	0.05	0.36
Geolocation		−1.30	−0.07	0.20
Parent education		1.88	0.11	0.06
*Step 2*	0.254			
Age *		2.02	0.10	0.04
Gender		0.06	0.00	0.95
Family income		0.10	0.01	0.92
Geolocation		−0.72	−0.04	0.47
Parent education *		2.07	0.10	0.04
Self-care efficacy ***		10.66	0.52	0.00

Note: * *p* < 0.05, *** *p* < 0.001.

**Table 5:** Hierarchical regression estimates predicting mental health in the face of schoolwork pressures.

Variable	Δ*R*^2^	*t*	*B*	*p*
*Step 1*	0.04			
Age		2.04	0.11	0.04
Gender *		−0.69	−0.04	0.49
Family income		1.79	0.10	0.07
Geolocation		−0.13	−0.01	0.90
Parent education		1.55	0.09	0.12
*Step 2*	0.21			
Age		1.22	0.06	0.22
Gender		0.15	0.01	0.99
Family income		1.17	0.06	0.24
Geolocation		0.54	0.03	0.59
Parent education		1.64	0.09	0.10
Self-care efficacy ***		9.19	0.46	0.00

Note: * *p* < 0.05, *** *p* < 0.001.

**Table 6:** Hierarchical regression estimates predicting self-care efficacy at T2.

Variable	Δ*R*^2^	*t*	*B*	*p*
*Step 1*	0.09			
Age		-0.53	−0.05	0.60
Gender *		−2.01	−0.18	0.05
Family income		1.68	0.17	0.10
Geolocation		1.60	0.15	0.11
Parent education		0.17	0.02	0.87
*Step 2*	0.19			
Age		−1.38	−0.11	0.17
Gender		-0.94	−0.08	0.35
Family income		0.99	0.09	0.33
Geolocation *		2.35	0.19	0.02
Parent education		0.96	0.09	0.34
Self-care efficacy at T1 ***		5.66	0.47	0.00
*Step 3*	0.04			
Age		−1.52	−0.12	0.13
Gender		−1.04	−0.08	0.30
Family income		0.87	0.08	0.39
Geolocation *		2.38	0.19	0.02
Parent education		0.90	0.08	0.37
Self-care efficacy at T1 ***		5.80	0.47	0.00
Practice frequency *		2.50	0.19	0.01

Note: * *p <* 0.05, *** *p <* 0.001.

**Table 7:** Differences between key variables in the experiment group at T1 and T2.

T2–T1	*M*	*SD*	*t*	*p*
Negative emotions **	−0.27	0.60	−2.71	0.009
Relationship-related mental health *	0.26	0.89	2.08	0.04
Schoolwork-related mental health **	0.29	0.78	2.76	0.008

Note: * *p* < 0.05, ** *p* < 0.01.

**Table 8:** Differences between key variables in the control group at T1 and T2.

T2–T1	*M*	*SD*	*t*	*p*
Negative emotions	−0.03	0.58	−0.25	0.80
Relationship-related mental health	0.10	0.62	1.30	0.20
Schoolwork-related mental health	0.04	0.62	0.59	0.56

## Data Availability

The data that support the findings of this study are available upon reasonable request from the corresponding author, [LX].

## Appendix A

**Table A1:** Measurements for key variables.

Variables and Items	Measurement Scale	Cronbach’s α
**Self-care efficacy**	1–5 Likert Scale	0.74
1. I have confidence in my ability to keep my stress within healthy limits.		
2. I have confidence in my ability to practice stress reduction techniques even when I’m stressed.		
3. I have confidence in my ability to help myself when I feel blue.		
4. I have confidence in my ability to maintain a positive attitude.		
5. I have confidence in my ability to experience life’s pleasures even when I’m stressed.		
6. I have confidence in my ability to cope with difficulties in my life.		
7. I have confidence in my ability to use techniques to manage my stress.		
**Negative emotions**	1–4 Likert Scale	0.89
1. I found it hard to wind down.		
2. I was aware of the dryness of my mouth.		
3. I couldn’t seem to experience any positive feelings at all.		
4. I experienced breathing difficulties (e.g., excessively rapid breathing, breathlessness in the absence of physical exertion).		
5. I found it difficult to work up the initiative to do things.		
6. I tended to overreact to situations.		
7. I experienced trembling (e.g., in the hands).		
8. I felt that I was using a lot of nervous energy.		
9. I was worried about situations in which I might panic and make a fool of myself.		
10. I felt that I had nothing to look forward to.		
11. I found myself getting agitated.		
12. I found it difficult to relax.		
13. I felt downhearted and blue.		
14. I was intolerant of anything that kept me from getting on with what I was doing.		
15. I felt I was close to panic.		
16. I was unable to become enthusiastic about anything.		
17. I felt I wasn’t worth much as a person.		
18. I felt that I was rather touchy.		
19. I was aware of the action of my heart in the absence of physical exertion (e.g., sense of heart rate increase, heart missing a beat).		
20. I felt scared without any good reason.		
21. I felt that life was meaningless.		
**Diener’s psychological well-being scale (measurements for both relationship- and schoolwork-related mental health)**	1–5 Likert Scale	0.83 for relationships;
1. I still lead a purposeful and meaningful life despite relationship/schoolwork stress.		0.88 for schoolwork
2. My overall social relationships are supportive and rewarding during situations like this.		
3. I am still engaged and interested in my daily activities during situations like this.		
4. I actively contribute to the happiness and well-being of others during situations like this.		
5. I am still competent and capable in the activities that are important to me.		
6. I am still a good person and live a good life.		
7. I am optimistic about my future.		
8. People still respect me.		
